# Longitudinal trajectories of emotional problems and unmet mental health needs among people newly diagnosed with HIV in China

**DOI:** 10.1002/jia2.25332

**Published:** 2019-08-19

**Authors:** Lu Niu, Dan Luo, Xi Chen, Min Wang, Wei Zhou, Dexing Zhang, Shuiyuan Xiao

**Affiliations:** ^1^ Department of Social Medicine and Health Management Xiangya School of Public Health Central South University Changsha China; ^2^ The Affiliated Brain Hospital of Guangzhou Medical University (Guangzhou Huiai Hospital) Guangzhou China; ^3^ Hunan Provincial Center for Disease Prevention and Control Changsha China; ^4^ HIV/AIDS Research Institute The First Hospital of Changsha Changsha China; ^5^ Hospital Administration Institute Xiangya Hospital Central South University Changsha China; ^6^ The Jockey Club School of Public Health and Primary Care Faculty of Medicine The Chinese University of Hong Kong Hong Kong China

**Keywords:** depression, anxiety, mental health services, HIV, China, longitudinal study

## Abstract

**Introduction:**

Concern over mental health morbidity affecting people living with HIV is increasing worldwide. The objective of this study was to describe the longitudinal trajectories of depression and anxiety, and mental health service utilization among people newly diagnosed with HIV.

**Methods:**

This was an observational cohort study that enrolled people newly diagnosed with HIV consecutively and followed them for one year in Changsha, China. Socio‐demographic, clinical and psychological data were collected at the baseline and at one‐year follow‐up. Participants were recruited between March 1, 2013 and September 30, 2014. The final follow‐up was in September 30, 2015.

**Results:**

Among 557 people newly diagnosed with HIV enrolled at the baseline, 410 (73.6%) completed the one‐year follow‐up survey (median (interquartile range) age at follow‐up: 29 (25, 39) years; 376 men (91.7%)), and were included in the analysis. 39.3% and 30.2% of the 410 participants were screened with significant symptoms of depression and anxiety at baseline respectively. An overall drop in the prevalence of each condition was found at follow‐up, however, 10.5% and 6.1% of participants were found to have persistent depression and anxiety. The results of mixed‐effect models showed that bisexuality, homosexual transmission, other clinical symptoms (for example, not on antiretroviral therapy (ART)), non‐disclosure, higher levels of HIV/AIDS‐related stress, and lack of social support were associated with significant symptoms of depression and anxiety. One year after diagnosis, 8.3% had visited healthcare providers for emotional or psychological problems.

**Conclusions:**

Despite the obvious need people newly diagnosed with HIV in China rarely seek professional help. Integrating depression and anxiety screening and referral into HIV care settings is warranted.

## 1 Introduction

Mental health morbidity affecting people living with HIV is becoming an increasing concern worldwide 1, 2, 3 with depression and anxiety most prevalent 4, 5. At the end of September 2018 there were 849,602 people living with HIV in China of whom 41.5% were AIDS patients 6. It is estimated that more than 60% and 40% of people living with HIV in China exhibited symptoms of depression and anxiety respectively 5. Moreover, depression and anxiety have been linked to suboptimal HIV treatment outcomes, worse quality of life and increased mortality 7, 8, 9, 10.

Depression and anxiety change over time and studies examining such conditions at a single time point will not represent their course and progression 11. Although an overwhelming majority of cross‐sectional studies have demonstrated the prevalence and related factors of depression and anxiety among people living with HIV, the results have been fragmentary and inconsistent 12, 13, 14, 15. Findings of the few existing longitudinal studies have also been mixed 16, 17, 18, 19, 20, 21, 22. Some studies have found an increase in depression and anxiety over time 16, 17, 18 others have found the prevalence to decline 19 or remain stable 20, 21, 22. These conflicting results may be due to differences in the sample characteristics, particularly the different disease courses of HIV infection 4, 5.

Diagnosis of HIV infection can be a direct stressor for reactive depression and anxiety in people newly diagnosed with HIV 23, 24 and little is known about the patterns and trajectories of these conditions among newly diagnosed people 5. The purpose of this study was to describe the trajectories of significant depression and anxiety symptoms, to identify associated socio‐demographic, clinical and psychosocial factors and to examine the utilization of mental health services among newly diagnosed people in China over a one‐year period.

## 2 Methods

### 2.1 Study setting and samples

This was an observational cohort study that enrolled people newly diagnosed with HIV and followed them for one year in Changsha, provincial capital of Hunan Province in central south China. By the end of 2017 Hunan Province had over 40.6 thousand cumulative diagnosed HIV cases, and sexual contact has become the primary mode of transmission with over 90% of new infections in the province due to this type of contact. We looked at people newly diagnosed with HIV between March 1, 2013 and August 30, 2014, all over 18 years of age and who had lived in Changsha for over six months. Participants were enrolled when they visited Changsha Centers for Disease Control and Prevention (CDC) to obtain certification of HIV‐infection diagnosis. Baseline data were collected face‐to‐face within 30 days of diagnosis and this baseline survey took place between March 1, 2013 and September 30, 2014. Participants were contacted for the follow‐up survey one year after baseline assessment and all outcome assessments were completed by September 30, 2015. The majority of participants completed the follow‐up survey having visited the CDC or the First Hospital of Changsha for regular tests. Thirty‐five participants refused face‐to‐face interviews but completed the survey either online (24/35) or by telephone (11/35). Failure to follow up was recorded as death of participants, relocation, rejection and lost. ‘Lost’ included poor retention within HIV care systoms and unreachable via the contact information participants provided at baseline. Dependent variables (depression and anxiety) and explanatory variables (including socio‐demographics, HIV clinical information, HIV/AIDS‐related stress and social support) were collected at both baseline and follow‐up. Mental health utilization throughout the year following diagnosis was also recorded at the follow‐up.

The study was approved by the Human Research Ethics Committee of Central South University (CTXY‐120033‐3), and all participants provided written informed consent. Participants were not compensated for taking part in the study.

### 2.2 Dependent variables

#### 2.2.1 Depression

Depression symptoms were assessed by the Patient Health Questionnaire Depression Scale (PHQ‐9) 25 which consists of nine criteria using a four‐point Likert‐type scale of how often a criterion has been a cause of emotional difficulty over a two week period, ranging from ‘0’ (not at all) to ‘3’ (nearly every day) 25. A score of 10 is usually the determinant for significant symptoms of depression 25 and the method has shown good reliability and legitimacy among Chinese people living with HIV 26, 27. In this study, the internal consistency estimate (Cronbach's α) was 0.913.

#### 2.2.2 Anxiety

Anxiety symptoms were assessed using the Generalized Anxiety Disorder Scale (GAD‐7) 28. This is similar to PHQ‐9 but consists of 7 criteria measured over the same time‐scale 28. A score of 10 points or higher is usually considered as indicating significant symptoms of anxiety 28. This too has shown itself to be a reliable and legitimate indicator in this study 26, 27. In this study, the value of Cronbach's α was 0.943.

#### 2.2.3 Mental health services utilization

At the one‐year visit participants were asked whether they had used health services specializing in emotional or psychological conditions. Those who answered “yes” were asked which institution they visited (e.g. specialty hospital, general hospital, CDC, psychological clinic).

### 2.3 Explanatory variables

#### 2.3.1 General information

Socio‐demographic characteristics were collected at baseline, these included gender, age, registered area of residence (i.e., urban or rural), marital status, educational background, employment status, average monthly family income, living alone or co‐habiting and sexual identity.

At the one‐year follow‐up visit participants were asked whether they had participated in peer support groups and self‐disclosed their HIV‐infection. If they had they were asked what type of group (spouses, friends, etc.).

#### 2.3.2 HIV clinical information

HIV clinical information included means of infection (sexual, blood transfusion and maternal‐neonatal), CD4 lymphocyte count (cells/μL), and additional HIV‐related symptoms such as persistent fevers for over one month, persistent diarrhoea or watery stools for over one month, persistent cough for over one month, over 10% weight loss in the last three months, thrush, recurrent herpes simplex, active tuberculosis and other symptoms as indicated by the participants. This information was collected at both the baseline and the one‐year visit. The treatment information (whether on ART or not) was collected via the CDC records at the one‐year visit.

#### 2.3.3 HIV/AIDS‐related stress

HIV/AIDS‐related stress was assessed by the Chinese version of the HIV/AIDS Stress Scale 29, 30. It consists of three subscales with 17 criteria; emotional stress, social stress and instrumental stress 30. Participants rated their perception of how distressing each challenging criteria was in the past month on a five‐point Likert scale, with higher scores indicating higher levels of stress. Acceptable reliability was shown in the present study with a Cronbach's α of 0.899.

#### 2.3.4 Social support

The Social Support Rating Scale is a 10‐criteria measure to assess social support in three major domains; objective social support, subjective social support and utility of social support 31. Higher scores suggest higher levels of social support, and in this study the Cronbach's α was 0.718.

### 2.4 Statistical analysis

First, descriptive statistics and frequencies were determined for all socio‐demographic, clinical and psychosocial outcomes (depression, anxiety, HIV/AIDS‐related stress and social support). We compared the distribution of all socio‐demographic, clinical and psychosocial characteristics at the baseline between lost samples and those retained at the one‐year visit using chi‐square tests or Mann‐Whitney U tests.

Secondly, we conducted paired Wilcoxon signed‐rank tests to compare the psychosocial outcomes at both the baseline and one‐year visits, as these variables were not normally distributed based on Kolmogorov‐Smirnov tests.

Thirdly, we used mixed‐effects logistic models 32 (with repeated measurements nested in individuals) to estimate the size of clinical (sexual orientation, transmission mode, number of symptoms, CD4 count, whether on ART or not) and psychosocial factors (living alone or not, disclosure of HIV‐infected status, participation at peer support groups, social support and HIV/AIDS‐related stress) associated with depression and anxiety. The variables used to determine whether or not participants were presenting significant symptoms of depression and anxiety were PHQ‐9, GAD‐7 >10 and PHQ‐9, GAD‐7<10 respectively. Univariate analyses were first used to assess the unadjusted odds ratios for each variable. Although there were divergence results about socio‐demographic differences of depression and anxiety in previous studies, most reported a significant association with gender, age, residence, marital status and socioeconomic status (which is usually measured by education, employment and income) in people living with HIV and those who are not 5, 33, 34, 35. Thus, we adjusted the crude odds ratios for each clinical and psychosocial factor to account for these variables and the residual pseudo‐likelihood technique was used for parameter estimation. Descriptive analyses were performed using SPSS version 17.0 (SPSS, Inc., Chicago, IL, USA) and mixed‐effects logistic regression model analyses were performed using Stata version 12.0 (StataCorp LP, College Station, TX, USA). A significance level of 0.05 was applied.

## 3 Results

### 3.1 Participant characteristics

From March 2013 to August 2014, 855 newly diagnosed HIV infections registered in the CDC system satisfied our inclusion criteria of whom 557 (65.1%) completed the questionnaire and were included in this study. An attrition rate of 26.4% occurred in the cohort. Reasons for attrition were death 2 (1.4%), transferred care centre 35 (23.8%), lost to follow‐up 35 (23.8%) and refusal to participate in the follow‐up survey 75 (51.0%). Table 1 summarizes the socio‐demographic characteristics. Lost samples were more likely to be employed than non‐lost samples (78.9% vs. 67.3%, *p *=* *0.015). Apart from employment, there were no notable differences in socio‐demographic, clinical or psychosocial characteristics at the baseline between lost samples and those retained at the one‐year visit.

**Table 1:** Sample characteristics and attrition analysis

	Baseline Total (N = 557)	One‐year follow‐up			
		Loss (n = 147)	Nonloss (n = 410)	χ^2^/μ	*p*
Gender				1.261	0.362
Male	515 (92.5)	139 (94.6)	376 (91.7)		
Female	42 (7.5)	8 (5.4)	34 (8.3)		
Age (median (IQR))^a^	28 (24, 37)	28 (24, 39)	28 (24, 36.25)	−0.560	0.575
Residence				0.105	0.773
Urban	283 (50.8)	73 (49.7)	210 (51.2)		
Rural	274 (49.2)	74 (50.3)	200 (48.8)		
Marital status				3.677	0.159
Single	347 (62.3)	95 (64.6)	252 (61.5)		
Married	139 (25.0)	29 (19.7)	110 (26.8)		
Divorced/widowed	71 (12.7)	23 (15.6)	48 (11.7)		
Education				2.694	0.441
Primary school or below	31 (5.6)	10 (6.8)	21 (5.1)		
Junior high school	105 (18.9)	22 (15.0)	83 (20.2)		
High school	166 (29.8)	48 (32.7)	118 (28.8)		
College or above	255 (45.8)	67 (45.6)	188 (45.9)		
Employment				6.161	0.015
Employed	391 (70.2)	115 (78.9)	276 (67.3)		
Unemployed/retired/other	166 (29.8)	32 (21.8)	134 (32.7)		
Monthly family income^a^				0.754	0.432
≤3500	271 (48.7)	67 (45.6)	204 (49.8)		
>3500	264 (47.4)	74 (50.3)	190 (46.3)		
Living alone				3.935	0.055
Yes	158 (28.4)	51 (34.7)	107 (26.1)		
No	399 (71.6)	96 (65.3)	303 (73.9)		
Sexual orientation				0.269	0.874
Heterosexuality	203 (36.4)	51 (34.7)	152 (37.1)		
Homosexuality	235 (42.2)	64 (43.5)	171 (41.7)		
Bisexuality	119 (21.4)	32 (21.8)	87 (21.2)		
Transmission mode^a^				2.868	0.238
Heterosexual	225 (40.4)	53 (36.1)	172 (42.0)		
Homosexual	321 (57.6)	92 (62.2)	229 (55.9)		
Non‐sexual	9 (1.6)	1 (0.7)	8 (2.0)		
CD4 count (cells/μL)^a^				0.005	0.945
≤350	262 (47.0)	65 (44.2)	197 (48.0)		
>350	281(50.4)	69 (46.9)	212 (51.7)		
HIV‐related symptoms				1.710	0.229
Without	358 (64.3)	101 (68.7)	257 (62.7)		
With	199 (35.7)	46 (31.3)	153 (37.3)		
HIV‐related stress (median (IQR))	21 (12.5, 31)	21 (12, 30)	21 (13, 32)	0.855	0.393
Social support (median (IQR))	29 (23, 34)	30 (22, 34)	29 (24, 34)	0.186	0.852
PHQ‐9 (median (IQR))	7 (3, 13)	6 (3, 13)	8 (3, 13)	1.025	0.305
Significant depression symptoms				1.270	0.277
Without (<10)	346 (62.1)	97 (66.0)	249 (60.7)		
With (≥10)	211 (37.9)	50 (34.0)	161 (39.3)		
GAD‐7 (median (IQR))	6 (2, 11)	6 (2, 10)	6 (3, 11)	0.578	0.563
Significant anxiety symptoms				0.051	0.916
Without (<10)	390 (70.0)	104 (70.7)	286 (69.8)		
With (≥10)	167 (30.0)	43 (29.3)	124 (30.2)		

GAD‐7, Generalized Anxiety Disorder‐7; IQR, interquartile range; PHQ‐9, Patient Health Questionnaire‐9.

a At baseline, age, monthly family income, transmission mode and CD4 count had missing values. The response rates of these factors were 98.2%, 96.1%, 99.6% and 97.4% respectively.

Data on the 410 participants who completed both baseline and follow‐up surveys were included in the analysis. At baseline, 37.3% on the participants reported HIV‐related symptoms, the mean CD4 count was 368.36 cells/μL (SD 186.43) with 48.0% having counts lower than 350 cells/μL. At the one‐year visit 27.1% of the participants reported HIV‐related symptoms the mean CD4 count was 423.93 cells/μL (SD 192.00) with 32.7% lower than 350 cells/μL. According to the CDC records, at the one‐year visit, 53.2% of the participants were on ART.

As shown in Table 2 scores of overall HIV‐related stress and the subscales declined by approximately 40% to 50% at the one‐year follow up. Scores of overall social support and objective support declined by approximately 10% to 20%. There was no significant statistically difference in subjective support and support utilization between baseline and follow‐up (*p *>* *0.05). Additionally, one year after diagnosis, 28.0% had participated in HIV/AIDS peer‐support groups and 72.0% had disclosed their HIV‐infected status to others. Specifically, 49.3% had disclosed it to their families (spouses, children, or relatives), and 25.1% to friends.

**Table 2:** Longitudinal trajectories of psychosocial outcomes among 410 people newly diagnosed with HIV (median (interquartile range))

	Baseline	One‐year follow‐up	Z	*p*
PHQ‐9	8 (3, 13)	4 (1, 8)	−9.772	<0.001
GAD‐7	6 (3, 11)	3 (0, 6)	−9.674	<0.001
CSS‐HIV	21 (13, 32)	13 (6, 20.25)	−11.288	<0.001
Emotional stress	6 (3, 10)	3 (1, 5)	−10.318	<0.001
Social stress	11 (7, 16)	7 (4, 11.25)	−10.129	<0.001
Instrumental stress	4 (1, 7)	2 (0, 5)	−7.793	<0.001
Social support	29 (24, 34)	27 (22, 34)	−3.256	<0.001
Perceived support	14 (11, 19)	14 (10, 20)	−0.186	0.852
Objective support	8 (6, 10)	6 (5, 8)	−7.040	<0.001
Support utilization	6 (5, 7)	6 (5, 7)	−0.633	0.527

CSS‐HIV, Chinese version of the HIV/AIDS Stress Scale; GAD‐7, Generalized Anxiety Disorder‐7; PHQ‐9, Patient Health Questionnaire‐9.

### 3.2 Longitudinal trajectories of depression and anxiety

#### 3.2.1 Depression

The scores of PHQ‐9 decreased significantly from 8 (interquartile range (IQR): 3, 13) at baseline to 4 (IQR: 1, 8) at the one‐year follow‐up (Z =* *−9.772, *p *<* *0.001). At baseline 39.3% of the 410 participants had significant symptoms of depression (PHQ‐9 ≥ 10), compared with 16.1% at the one‐year follow‐up.

The trajectory of depression is presented in Table 3 and whereas 28.7% showed a pattern of remission during the one‐year follow‐up period, 10.5% had significant symptoms of depression at both baseline and follow‐up, and, 5.6% who were without depression at baseline showed significant symptoms of depression at follow‐up.

**Table 3:** Longitudinal trajectories of depression and anxiety among 410 people newly diagnosed with HIV (n/%)

Baseline	One‐year follow up	
	With	Without
Significant depression symptoms		
With (≥10)	43 (10.5)	118 (28.7)
Without (<10)	23 (5.6)	226 (55.2)
Significant anxiety symptoms		
With (≥10)	25 (6.1)	99 (24.1)
Without (<10)	25 (6.1)	261 (63.7)

#### 3.2.2 Anxiety

The scores on GAD‐7 decreased from 6 (IQR: 3, 11) at baseline to 3 (0, 6) at the one‐year follow‐up (Z =* *−9.674, *p *<* *0.001). At baseline 30.2% of the 410 participants had significant anxiety symptoms (GAD‐7 ≥ 10) compared with 12.2% at the one‐year follow‐up.

As shown in Table 3 whereas 24.1% showed a pattern of remission during the one‐year follow‐up period, 6.1% had positive anxiety symptoms at both baseline and follow‐up and 6.1% who were without anxiety at baseline showed significant anxiety symptoms at follow‐up.

### 3.3 Determinants of depression and anxiety over one year

A summary of the factors associated with significant depression and anxiety symptoms among participants is shown in Table 4.

**Table 4:** Factors associated with significant depression and anxiety symptoms in people newly diagnosed with HIV (n = 410)

Variables	Depression				Anxiety			
	uOR (95% CI)	*p*	aOR (95% CI)	*p*	uOR (95% CI)	*p*	aOR (95% CI)	*p*
Sexual orientation								
Heterosexuality	1.00		1.00		1.00		1.00	
Homosexuality	1.08 (0.74, 1.58)	0.681	1.47 (0.97, 2.23)	0.067	1.001 (0.67, 1.51)	0.974	1.20 (0.78, 1.86)	0.403
Bisexuality	1.38 (0.88, 2.145)	0.157	2.03 (1.29, 3.30)	0.002	1.35 (0.85)	0.209	1.67 (1.04, 2.68)	0.035
Transmission mode								
Heterosexual	1.00		1.00		1.00		1.00	
Homosexual	1.30 (0.93, 1.83)	0.130	1.65 (1.14, 2.39)	0.009	1.32 (0.91, 1.90)	0.139	1.50 (1.01, 2.23)	0.043
Non‐sexual	1.02 (0.29, 3.54)	0.975	1.23 (0.38, 3.93)	0.729	1.46 (0.43, 4.99)	0.549	1.16 (0.34, 3.95)	0.818
Symptom counts	1.73 (1.38, 2.18)	<0.001	1.78 (1.43, 2.21)	<0.001	1.60 (1.26, 2.03)	<0.001	1.63 (1.31, 2.03)	<0.001
CD4 counts	1.00 (0.99, 1.00)	0.345	1.00 (0.99, 1.01)	0.423	1.00 (0.99, 1.00)	0.947	1.00 (0.99, 1.01)	0.916
On ART								
Yes	1.00		1.00		1.00		1.00	
No	3.03 (1.98, 4.65)	<0.001	3.21 (2.10, 4.91)	<0.001	2.66 (1.67, 4.24)	<0.001	2.90 (1.85, 4.69)	<0.001
Living alone								
No	1.00				1.00		1.00	
Yes	0.62 (0.42, 0.92)	0.018	0.66 (0.46, 0.95)	0.024	0.84 (0.56, 1.27)	0.415	0.89 (0.62, 1.31)	0.569
Self‐help peer groups								
Yes	1.00		1.00		1.00		1.00	
No	0.63 (0.33, 1.22)	0.169	0.64 (0.37, 1.12)	0.113	0.98 (0.46, 2.09)	0.966	0.99 (0.53, 1.86)	0.972
Disclosure								
Yes	1.00		1.00		1.00		1.00	
No	3.23 (2.21, 4.71)	<0.001	3.30 (2.27, 4.79)	<0.001	2.53 (1.69, 3.78)	<0.001	2.73 (1.83, 4.07)	<0.001
Subjective social support	0.95 (0.92, 0.97)	<0.001	0.94 (0.91, 0.96)	<0.001	0.96 (0.93, 0.98)	0.003	0.96 (0.93, 0.98)	0.002
Objective social support	0.90 (0.86, 0.95)	<0.001	0.89 (0.85, 0.94)	<0.001	0.87 (0.81, 0.92)	<0.001	0.85 (0.81, 0.90)	<0.001
Support utilization	0.96 (0.88, 1.04)	0.276	0.94 (0.87, 1.02)	0.124	0.93 (0.84, 1.01)	0.068	0.92 (0.84, 0.99)	0.037
Social stress	1.21 (1.17, 1.25)	<0.001	1.22 (1.19, 1.26)	<0.001	1.22 (1.17, 1.26)	<0.001	1.23 (1.19, 1.27)	<0.001
Instrumental stress	1.31 (1.24, 1.37)	<0.001	1.31 (1.25, 1.37)	<0.001	1.29 (1.23, 1.36)	<0.001	1.29 (1.23, 1.35)	<0.001

aOR, adjusted odds ratios, adjusted for gender, age, residence, marital status, educational background, employment, monthly family income; ART, antiretroviral therapy; CI, confidence interval; uOR, unadjusted odds ratios.

Factors found to be associated with depression were: bisexuality (adjusted odds ratios (aOR) 2.03; 95% CI 1.29, 3.30), homosexual transmission (aOR 1.65; 95% CI 1.14, 2.39), number of HIV‐related symptoms (aOR 1.78; 95% CI 1.43, 2.21), not on ART (aOR 3.21; 95% CI 2.10, 4.91), living alone (aOR 0.66; 95% CI 0.46, 0.95), non‐disclosure (aOR 3.30; 95% CI 2.27, 4.79), subjective social support (aOR 0.94; 95% CI 0.91, 0.96), objective social support (aOR 0.89; 95% CI 0.85, 0.94), HIV/AIDS related social stress (aOR 1.22; 95% CI 1.19, 1.26) and HIV/AIDS related instrumental stress (aOR 1.31; 95% CI 1.25, 1.37). People newly diagnosed with HIV who were bisexual infected via homosexual activities, living with others, had not disclosed HIV status to others, had more HIV‐related symptoms and had higher levels of HIV/AIDS related social and instrumental stress, but lower levels of subjective and objective social support, were more likely to lead to significant symptoms of depression.

Factors associated with anxiety symptom were; bisexuality (aOR 1.67; 95% CI 1.04, 2.68), homosexual transmission (aOR 1.50; 95% CI 1.01, 2.23), number of HIV‐related symptoms (aOR 1.63; 95% CI 1.31, 2.03), not on ART (aOR 2.90; 95% CI 1.85, 4.69), non‐disclosure (aOR 2.73; 95% CI 1.83, 4.07), subjective social support (aOR 0.96; 95% CI 0.93, 0.98), objective social support (aOR 0.85; 95% CI 0.81, 0.90), social support utilization (aOR 0.92; 95% CI 0.84, 0.99), HIV/AIDS related social stress (aOR 1.23; 95% CI 1.19, 1.27) and HIV/AIDS related instrumental stress (aOR 1.29; 95% CI 1.23, 1.35). People newly diagnosed with HIV who were bisexual, infected via homosexual activities, had not disclosed HIV status to others, had more HIV‐related symptoms, and had higher levels of HIV/AIDS related social and instrumental stress, but lower levels of subjective and objective social support, and social support utilization, were more likely to lead to significant anxiety symptoms.

### 3.4 Mental health services utilization during one year after being diagnosed

**Figure 1:**
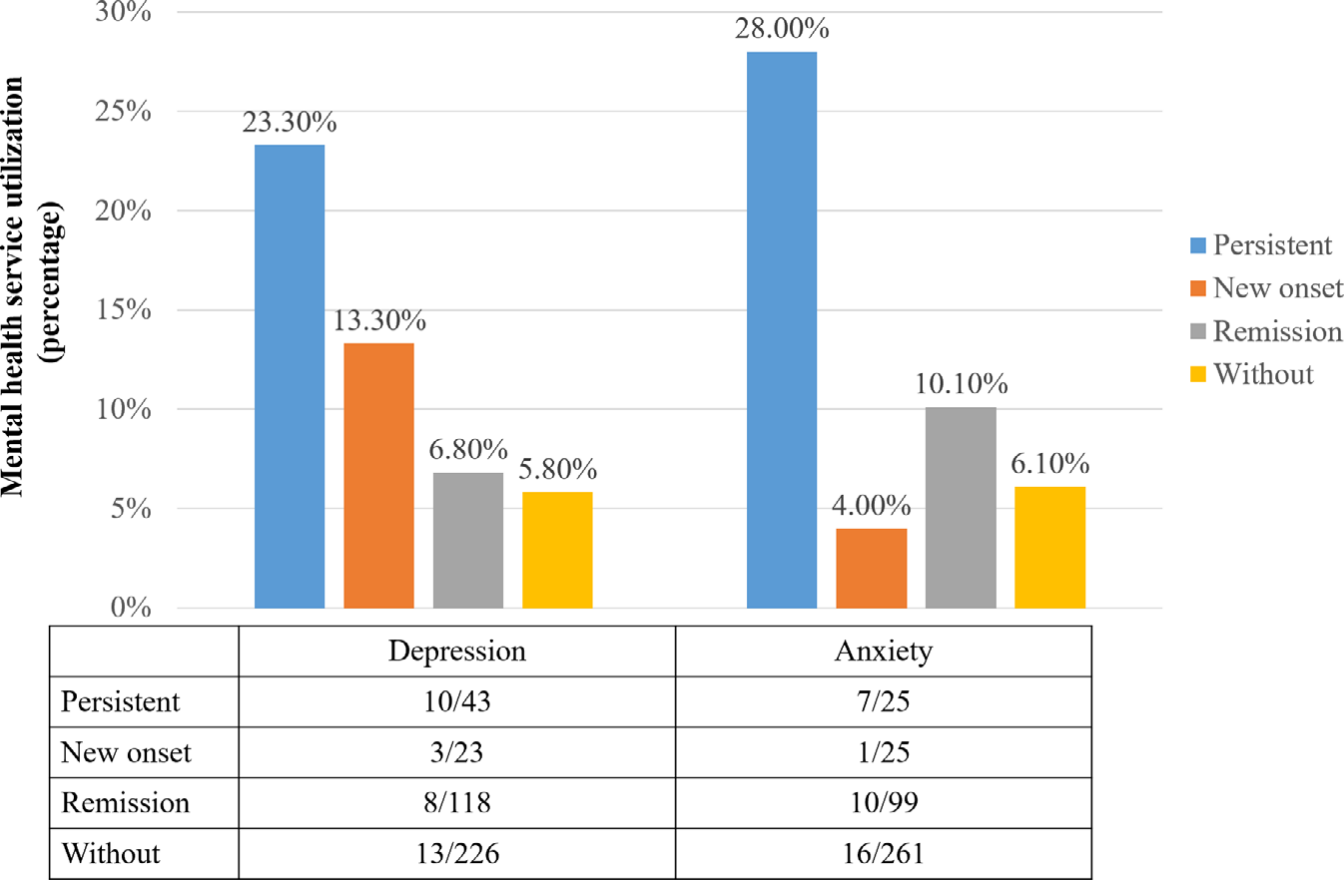
Mental health service utilization among people newly diagnosed with HIV (n = 410)

One year after diagnosis, 8.3% (34/410) of participants had visited doctors/healthcare providers for emotional or psychological problems, of whom 17 specified the provider they visited. One visited a psychology consulting institution, three sought help from the CDC staff and the remainder went to [general] hospitals for help without identifying the departments they visited. As shown in Figure 1, participants who had persistent depression or anxiety were more likely to visit doctors/healthcare providers for emotional or psychological problems (depression: χ^2^ **= 15.615, *p *=* *0.001; anxiety: χ^2^ **= 15.403, *p *=* *0.002).

## 4 Discussion

This study describes trajectories of depression and anxiety in a cohort of people newly diagnosed with HIV in Changsha, south central China. We found high prevalence of symptoms of depression and anxiety measured just after HIV diagnosis and an overall drop in the prevalence of each condition from baseline to follow‐up. We also identified a large percentage of people who had significant depression and anxiety symptoms at both visits, suggesting the persistence of emotional problems. Continued mental health support and prevention services, especially at early stage, are, therefore, warranted.

Despite the obvious need, use of mental health services was low, even among those with persistent depression or anxiety. Similarly, in a study of 28 Chinese people living with HIV Jin *et al*. 36 found that of the 22 participants diagnosed with lifetime major depressive disorders only 9% had received treatment. Wu *et al*. 37 found that among 175 HIV infections in China 14.5% had seen a doctor for psychological problems, 12 participants had attempted suicide and only three of those had seen a doctor before suicide attempts.

We can reasonably assume that gender composition is a reason for the low rates of mental health service usage as the majority of our participants were male. Other research has indicated that men utilize mental health services less frequently than women due to masculine gender socialization 38. Furthermore, the fear of discrimination may also explain low mental health service usage 39, 40, 41.

Integration of screening and managing mental health in the HIV care setting has been identified as a promising strategy to improve mental health and HIV treatment outcomes among people living with HIV in low‐ and middle‐income countries 42. However, significant gaps exist in health services in China. First, there is neither sufficient screening nor evidence‐based guidelines for mental health management in HIV care settings. Secondly, a lack of referral also highlights a current disadvantage in HIV care. Strategies to increase access to mental health services in HIV care settings should be explored.

Being infected with HIV is an extremely stressful experience that affects almost every aspect of a person’s life 43, 44. Consistent with previous studies 45, 46, 47 we found that people with higher HIV‐related stress were more likely to have symptoms of significant depression and anxiety. The study results of HIV‐related stress had significantly declined at the one‐year follow‐up and consequently the levels of depression and anxiety had declined. According to Lazarus and Folkman’ s conceptualization of stress and coping 48, the extent to which an event is perceived as stressful largely depends on the person’ s appraisal of the event regarding its potential harm, controllability and mastery. Our results indicate that people could better cope with their HIV infection across time, however, HIV infection is a persistent stressor and it requires regular evaluation and constant psychosocial support for stress management 30.

Current literature shows social support is associated with a lower probability or level of depression and anxiety in people living with HIV 47, 48, 49, 50. We also found that social support was a protective factor of depression and anxiety and an additional major finding was a decline in social support at one year after diagnosis. Two possible explanations are (1) problems related to social stress were most troublesome for people living with HIV 29, 30. Thus, after diagnosis people may choose to reduce social contact and interaction; (2) over two‐thirds of the participants had disclosed their HIV‐infection status to others who might themselves be afraid of infection or discriminatory attitudes toward them regarding HIV infection, resulting in loss of social support 49.

Furthermore, we found that bisexuality and being infected through homosexual behaviours were more likely to result in significant symptoms of depression and anxiety. Disparities in depression and anxiety between heterosexual and sexual minority individuals have been overwhelmingly reported in both the general population and people living with HIV 50, 51, 52. Men who have sex with men have accounted for 20% to 28% of new infections in China each year since 2013 6, 53, 54, 55, 56, 57 and mental health support programme should give more consideration to HIV infections among this vulnerable population.

Consistent with previous studies 4, 19, 27 we found that the number of HIV‐related symptoms had a substantial impact on the probability of screening positive for significant symptoms of depression and anxiety. HIV‐related symptoms may be a constant and notable reminder, and many symptoms can be painful and debilitating, resulting in higher levels anxiety or depression, or both 19, 58. Moreover, people who did not start ART in the follow‐up year were three times more likely to present significant symptoms of depression and anxiety. These findings suggest palliation of HIV‐related symptoms and early ART initiation are important considerations for the well‐being of people living with HIV.

A paradoxical finding was identified with a lower probability of significant symptoms of depression or anxiety among those living alone. A possible explanation is that people who live alone have less social stress related to disclosure, infection and privacy concerns and thus have lower levels of depression and anxiety at the early stage of HIV infection 59. As documented in numerous studies, social support is an important protective factor not only for mental health but also for treatment adherence and physical health outcomes 60, 61, 62, 63, 64. Living alone could be a risk factor for people with HIV long‐term due to the lack of social connectedness 59 and it is worth examining the long‐term impact on mental health of people living alone with HIV.

This study had several limitations. First, although the present study was based on a consecutive sample in Changsha, the sample has a certain typicality as the characteristics were consistent with the national report (i.e. the majority of people newly diagnosed with HIV were male and infected through homosexual behaviours) 6. Secondly, we did not measure premorbid depression or anxiety prior to participants receiving HIV diagnoses. Thirdly, reporting bias may exist due to the nature of self‐reported data. Fourthly, the trajectory over one year was a relatively short period in terms of the whole course of HIV/AIDS, however, we are still following these participants and we will continue focusing on the trajectories of depression and anxiety over a longer period and their association with the course of the disease.

## 5 Conclusions

This report provides new insights into the longitudinal trajectories of depression and anxiety among people living with HIV. Depression and anxiety are highly prevalent particularly among those newly diagnosed. Although the severity of depression and anxiety seems to decrease across time a considerable proportion of newly diagnosed people have persistent depression and anxiety. Despite the obvious need newly diagnosed people in China, even those with persistent depression and anxiety, rarely seek professional help and programmes are warranted to integrate depression and anxiety screening and referral to HIV care settings. Future research should develop evidence‐based guidelines for the screening and managing of emotional problems and explore strategies to increases access to mental health services in HIV care settings. Targeted intervention programmes are also required to help newly diagnosed people acquire stress management skills and obtain the social support they need to prevent and alleviate depression and anxiety.

## Competing interests

We declare no competing interests.

## Authors’ contributions

DL and SX conceived and designed the study, were responsible for study coordination and data management, assisted in interpretation and manuscript writing. LN contributed to study design, analysed data and wrote the manuscript. XC and MW assisted in reviewing protocol and study coordination in the field, and reviewed the manuscript. WZ and DZ critically reviewed the manuscript for important intellectual content. All authors have read and approved the final manuscript.

## Funding

This study was supported by the National Natural Science Foundation of China (81202290) and the U.S. National Institutes of Health (D43 TW009101).
